# Molecular Characterization of Virulence Genes and Quinolone Resistance Determinants in *Salmonella* Strains from Retail Chicken Meat

**DOI:** 10.3390/antibiotics15080776

**Published:** 2026-08-12

**Authors:** Hüsnü Şahan Güran, Nurdan Karacan Sever, Bülent Bozdoğan, Resat Çiftçi, Walid Alali, Yunus Doğan, Uğur Uçar

**Affiliations:** 1 Department of Food Hygiene and Technology, Faculty of Veterinary Medicine, Dicle University, Diyarbakır 21280, Türkiye; 2 Department of Microbiology, Faculty of Veterinary Medicine, Dicle University, Diyarbakır 21280, Türkiye; 3 Department of Medical Microbiology, Faculty of Medicine, Aydin Adnan Menderes University, Aydın 09100, Türkiye; 4 Batman Directorate of Provincial Agriculture and Forestry, Republic of Türkiye Ministry of Agriculture and Forestry, Batman 72070, Türkiye; 5 Department of Biostatistics and Epidemiology, College of Public Health, East Tennessee State University, Johnson City, TN 37614, USA; 6 Department of Biotechnology, Experimental Medicine Research and Application Center, University of Health Sciences, İstanbul 34668, Türkiye

**Keywords:** *Salmonella*, quinolone resistance determinants, colistin resistance, virulence genes, class 1 integron, chicken meat

## Abstract

**Background/Objectives:** *Salmonella* is a major foodborne pathogen commonly associated with poultry products and poses a significant public health concern worldwide. This study aimed to determine the distribution of *Salmonella* serotypes isolated from retail chicken meat and to evaluate their quinolone resistance profiles, along with selected virulence and antimicrobial resistance genes. **Methods:** A total of 53 *Salmonella* isolates recovered from retail chicken meat were serotyped and subjected to quinolone susceptibility testing and PCR-based detection of class 1 integrons, antimicrobial resistance genes, and virulence-associated genes. **Results:** Of these, 41 (77.35%) were identified as *Salmonella* Infantis (*S.* Infantis) and 12 (22.65%) as *Salmonella* Enteritidis (*S.* Enteritidis). Antimicrobial susceptibility testing revealed that 47 (88.67%) isolates were resistant to enrofloxacin, 32 (60.37%) to ciprofloxacin, 26 (49.05%) to moxifloxacin, and 24 (45.28%) to norfloxacin. All *S.* Enteritidis isolates were susceptible to ciprofloxacin, moxifloxacin, and norfloxacin. Broth microdilution results showed that 20.75% of isolates were susceptible and 77.35% were intermediate to ciprofloxacin, with minimum inhibitory concentration values ranging between 0.02 and 1 µg/mL. The class 1 integron gene (*int1*) was detected in 83.01% of isolates. The *mcr1*, *mcr2*, *mcr3*, *mcr4*, *mcr5*, *qnrA*, and *qnrC* genes were not detected in any isolates. Among *S.* Infantis isolates, 2 (4.88%) and 9 (21.95%) were positive for *qnrB* and *qnrSm* genes, respectively, while all *S.* Enteritidis isolates were negative for these genes. Virulence gene analysis showed that *sitC* was detected in 100% of isolates and *sopE* in 98.11%. The *spvC* and *pefA* genes were detected in 13.2% of isolates. **Conclusions:** The findings suggest that *S.* Infantis was the predominant serotype and that quinolone resistance was common among *Salmonella* isolates from retail chicken meat. The virulence gene profiles indicated that these isolates harbored a wide range of markers associated with host invasion, intracellular survival, and adaptation within the host environment. Moreover, the high prevalence of the *int1* gene highlights the need for further investigation of integron-associated resistance determinants and their potential role in the dissemination of antimicrobial resistance through chicken products and the associated risk of transmission to humans.

## 1. Introduction

*Salmonella* enterica remains one of the leading causes of foodborne bacterial gastroenteritis worldwide and represents a major public health concern within the One Health framework [1,2]. More than 2600 serovars of *Salmonella* enterica have been described; however, only a relatively small number of these serovars are responsible for the majority of human and animal infections. Moreover, the distribution of predominant serovars is dynamic and varies over time, geographic region, host species, and ecological niche [3]. Changes in poultry production systems, together with antimicrobial use practices and the emergence of successful epidemic clones, have contributed to shifts in the epidemiology and predominance of *Salmonella* serovars in many countries [4,5,6].

Antimicrobial resistance is recognized as one of the greatest global public health challenges. The widespread use of antimicrobials in both human and veterinary medicine has accelerated the emergence of resistant *Salmonella* strains, thereby limiting therapeutic options for invasive infections [7,8]. Fluoroquinolones represent a cornerstone in the management of invasive salmonellosis, with ciprofloxacin serving as both a principal therapeutic agent for severe infections and the definitive reference fluoroquinolone for *Salmonella* susceptibility testing in accordance with CLSI and EUCAST criteria [9,10]. However, the extensive use of fluoroquinolones in food-producing animals has contributed to the global emergence of quinolone-resistant *Salmonella*. Quinolone resistance develops through chromosomal mutations within the quinolone resistance-determining regions (QRDRs) of *gyrA*, *gyrB*, *parC*, and *parE*, as well as through the acquisition of plasmid-mediated quinolone resistance (PMQR) determinants, including *qnr* genes, *qepA*, *oqxAB*, and *aac(6′)-Ib-cr* [11,12,13,14,15]. In addition, class 1 integrons facilitate the capture and dissemination of antimicrobial resistance gene cassettes, thereby contributing to the persistence and spread of multidrug-resistant *Salmonella* populations in food animal production [16,17,18].

In recent years, plasmid-mediated colistin resistance has emerged as an important public health concern because colistin remains one of the last-line therapeutic options for treating severe infections caused by multidrug-resistant and carbapenem-resistant Gram-negative bacteria [19,20]. In *Salmonella*, colistin resistance is predominantly associated with chromosomal mutations that modify the lipid A component of lipopolysaccharide (LPS), thereby reducing the affinity of colistin for the bacterial outer membrane [21]. However, the discovery and global dissemination of mobilized colistin resistance (*mcr*) genes have raised concerns regarding transferable resistance mechanisms, as these genes can spread horizontally among members of the *Enterobacteriaceae* [22]. To date, multiple *mcr* gene families have been identified, exhibiting considerable geographic and ecological variation in their distribution [23,24].

The investigation of virulence gene repertoires is important for understanding the bacterial properties of *Salmonella* serovars. The virulence mechanisms of individual *Salmonella* serovars have not yet been fully elucidated [25]. Variations in virulence factor profiles among serovars are key determinants of pathogenicity, host specificity, and epidemiological success [26]. The ability of *Salmonella* which cause infection in humans and animals to neutralize the host’s defense and escape from the host is associated with the coding and expression of various virulence genes [26,27]. The virulence genes encode many effector proteins that play an important role in the pathogenesis of *Salmonella* [26]. Effector proteins such as sipA, sipD, sopB, sopD, sopE and sopE2, and fimbria encoded by *pefA* play a role in the adhesion and invasion of *Salmonella* into the host cell [4,27,28]. The *sitC* gene expressed from the *sitABCD* operon is required for proliferation and survival of *Salmonella* under conditions where there is not enough iron [29,30]. SsaR and sifA effector proteins are involved in many important phases such as invasion, internalization, intracellular proliferation and survival of *Salmonella* [4,30,31]. The *spv* operon carrying the *spvRABCD* genes is associated with the development of systemic infection and proliferation of *Salmonella* in the reticuloendothelial system [4,28].

Although *Salmonella* is a major foodborne pathogen of global public health significance, comprehensive data on the serotype distribution, quinolone susceptibility, virulence-associated genes, and molecular determinants of antimicrobial resistance among isolates recovered from retail chicken meat in Türkiye remain limited. Such information is essential for improving our understanding of the epidemiology, pathogenic potential, and antimicrobial resistance profiles of poultry-associated *Salmonella*, thereby supporting effective surveillance and control strategies. Accordingly, this study aimed to determine the serotype distribution of *Salmonella* isolates recovered from retail chicken meat, assess their phenotypic susceptibility to clinically relevant quinolone antimicrobials, and characterize molecular determinants associated with antimicrobial resistance, including plasmid-mediated quinolone resistance genes (*qnrA*, *qnrB*, *qnrC*, and *qnrSm*), transferable colistin resistance genes (*mcr1*, *mcr2*, *mcr3*, *mcr4*, and *mcr5*), class 1 integrons, together with virulence-associated genes (*invA*, *sipA*, *sipD*, *sopD*, *sopB*, *sopE*, *sopE2*, *sitC*, *ssaR*, *sifA*, *spvC*, and *pefA*).

## 2. Results

Among the 53 *Salmonella* isolates analyzed, *S*. Infantis was the predominant serotype (77.35%), followed by *S*. Enteritidis (22.65%). Quinolone resistance was frequently observed, with enrofloxacin showing the highest resistance rate, followed by ciprofloxacin, moxifloxacin, and norfloxacin (Table 1). Resistance to all four quinolone antimicrobials was significantly more common in *S*. Infantis than in *S*. Enteritidis (*p* < 0.05). Notably, all *S*. Enteritidis isolates were susceptible to ciprofloxacin, moxifloxacin, and norfloxacin.

Six isolates (one *S*. Infantis and five *S*. Enteritidis) were susceptible to all tested quinolone antimicrobials. The remaining 47 resistant isolates were classified into seven distinct quinolone resistance patterns, as summarized in Table 2.

Resistance pattern analysis showed that most *S*. Infantis isolates belonged to patterns A and B (Table 2). Ciprofloxacin MIC testing by the broth microdilution method showed that 20.75% of the isolates were susceptible, whereas 77.35% exhibited intermediate susceptibility (MICs: 0.02–1 µg/mL) (Table 3). Among *S*. Infantis isolates, 97.56% were classified as intermediate and only one isolate (2.44%) was resistant to ciprofloxacin. In contrast, no ciprofloxacin-resistant *S*. Enteritidis isolates were detected. 

The *int1* gene was detected in 44 (83.01%) of the *Salmonella* isolates, with no significant difference in its distribution between *S.* Infantis and *S*. Enteritidis (*p* > 0.05) (Table 4). None of the isolates carried the investigated *mcr1–mcr5*, *qnrA*, or *qnrC* genes. Among the plasmid-mediated quinolone resistance (PMQR) determinants, *qnrB* and *qnrSm* were detected exclusively in *S*. Infantis, with positivity rates of 4.88% and 21.95%, respectively, whereas all *S*. Enteritidis isolates were negative for these genes.

Virulence-associated genes were widely distributed among the *Salmonella* isolates (Table 5). The *sitC* and *sopE* genes were detected in 100% and 98.11% of the isolates, respectively, whereas *spvC* and *pefA* were each detected in 13.2% of the isolates. The *sopE2*, *spvC*, and *pefA* genes were not detected in *S*. Infantis, while *sipD* was absent from all *S.* Enteritidis isolates. Significant differences in the distribution of several virulence genes were observed between the two serotypes. The frequencies of *sipD* and *sopB* were significantly higher in *S*. Infantis, whereas *sopE2*, *spvC*, and *pefA* were significantly more prevalent in *S*. Enteritidis (*p* < 0.05). In contrast, no significant differences were observed in the distribution of *sipA*, *sopD*, *sitC*, *ssaR*, *sifA*, or *sopE* between the two serotypes (*p* > 0.05).

Analysis of virulence gene profiles identified 27 distinct profiles among the 53 *Salmonella* isolates, including 18 profiles in *S*. Infantis (Table 6). Most isolates belonged to virulence profiles 1, 2, and 3, with these profiles predominating among *S.* Infantis.

## 3. Discussion

Our study revealed that *S.* Infantis was the predominant serotype among the *Salmonella* isolates recovered from retail chicken meat, whereas *S.* Enteritidis was less prevalent. This finding is similar to the increasing global trend in *S.* Infantis detection in broiler poultry production and poultry meat. Several studies have reported that *S.* Infantis has emerged as a frequently detected serotype in poultry production and has been linked to multidrug-resistant infection in humans. Interestingly, *S.* Infantis has shown the ability to persist in poultry production environments. Additionally, it can acquire pESI-like megaplasmids that can enhance bacterial fitness, persistence, and antimicrobial resistance [4,5,6,32,33]. 

A main finding from this study was the high prevalence of quinolone resistance, especially among *S.* Infantis isolates. This finding is significant for public health because fluoroquinolones are key antibiotics used to treat invasive salmonellosis. Resistance to quinolone and fluoroquinolone at high levels in *Salmonella* from poultry production systems have been frequently reported and suggested to be associated with selection pressure due to antibiotic use in food animal production [7,8,17,34]. The higher frequency of quinolone resistance observed in *S.* Infantis compared with *S.* Enteritidis may reflect differences in the acquisition and maintenance of antimicrobial resistance determinants, including mobile genetic elements, between these serovars. This observation is consistent with previous studies describing the emergence and dissemination of quinolone-resistant *S.* Infantis in poultry production [5,6,35,36,37]. 

This study showed a discrepancy between ciprofloxacin susceptibility results obtained by disk diffusion and broth microdilution. Although several isolates were categorized as resistant by disk diffusion, they exhibited intermediate ciprofloxacin MIC values in the broth microdilution assay, suggesting reduced ciprofloxacin susceptibility rather than high-level fluoroquinolone resistance. Such discrepancies may reflect methodological differences between disk diffusion and broth microdilution, particularly for *Salmonella*. According to the European Committee on Antimicrobial Susceptibility Testing [10], testing with a 5 μg ciprofloxacin disk does not reliably exclude all fluoroquinolone resistance mechanisms in *Salmonella* spp., and ciprofloxacin MIC determination is therefore recommended. This discrepancy highlights the complexity of quinolone resistance in *Salmonella*. Fluoroquinolone resistance is primarily associated with chromosomal mutations within the quinolone resistance-determining regions (QRDRs) of *gyrA*, *gyrB*, *parC*, and *parE*, whereas plasmid-mediated quinolone resistance (PMQR) genes generally confer only low-level resistance and facilitate the selection of higher levels of fluoroquinolone resistance [11,12,13,38]. In the present study, *qnrB* and *qnrSm* were detected at a low frequency, indicating that these genes alone are unlikely to account for the observed phenotypic resistance. Therefore, the discrepancy between the phenotypic and genotypic findings suggests that additional resistance mechanisms, particularly chromosomal QRDR mutations and possibly efflux-associated mechanisms, contributed to the quinolone-resistant phenotype observed in these isolates [13].

An important finding of the present study was the high prevalence of the class 1 integron integrase gene (*int1*) among our isolates. Class 1 integrons are well-known mobile genetic elements involved in the acquisition and expression of antimicrobial resistance gene cassettes and have been linked to multidrug-resistant *Salmonella* spp. and other members of the family *Enterobacteriaceae* [16]. The high prevalence of the *int1* gene observed in this study indicates the widespread presence of class 1 integrons among the *Salmonella* isolates. Although class 1 integrons are commonly associated with the acquisition and dissemination of antimicrobial resistance determinants, the present study did not characterize integron gene cassette arrays, associated resistance genes, or the genetic location of the integrons. Therefore, their role in the dissemination of antimicrobial resistance could not be determined based on the present findings. Despite the limited number of antimicrobial resistance genes investigated in the present study, the high prevalence of class 1 integrons highlights the importance of further characterization of integron gene cassettes and associated resistance determinants in *Salmonella* from poultry production [17,18].

Interestingly, *mcr1*, *mcr2*, *mcr3*, *mcr4*, and *mcr5* were not detected in any of the *Salmonella* isolates. Since phenotypic susceptibility to colistin was not evaluated, these findings should be interpreted only with respect to the investigated transferable colistin resistance determinants rather than as evidence for the absence of colistin resistance. Although none of the investigated *mcr* genes were detected, colistin resistance in *Salmonella* may also arise through chromosomal mutations, and additional *mcr* variants beyond *mcr5* have been described [21,22,23]. Furthermore, the distribution of *mcr* genes varies according to geographic region, host species, and ecological niche [24]. Therefore, the absence of the investigated *mcr* genes should be interpreted with caution and does not exclude the presence of other transferable or chromosomally mediated mechanisms of colistin resistance.

The virulence gene profiles indicated that these isolates harbored a wide range of markers associated with host invasion, intracellular survival, and adaptation within the host environment. The universal presence of *sitC* and the high frequency of *sopE* suggest that most isolates possessed determinants associated with iron acquisition and epithelial cell invasion. Similarly, the frequent presence of *sipA*, *sipD*, *sopD*, and *sopB* in our isolates further implies the pathogenic potential of these *Salmonellae*. It has been reported that these genes are involved in invasion and modulation of host cell responses [4,27,30,39]. Collectively, these results suggest that retail chicken meat is a possible vehicle for *Salmonella* strains with substantial virulence potential.

Differences between serotypes were also observed in the virulence gene profiles. In this study, *sopE2*, *spvC*, and *pefA* were not found in *S.* Infantis. However, these genes were detected in a fraction of *S.* Enteritidis isolates. This finding is important because *spv* and *pef* genes are frequently linked with virulence plasmids as well as systemic infection potential and fimbrial adhesion in some *Salmonella* serovars [28,40]. Although *S.* Infantis was the major serotype in this study with higher quinolone resistance, the presence of *spvC* and *pefA* in *S.* Enteritidis may indicate additional plasmid-mediated virulence traits in this serotype.

Overall, the results of this study show that retail chicken meat can carry *Salmonella* strains with both quinolone resistance and several virulence genes. This is important to food safety and One Health because poultry meat may be a source of Salmonella strains that can spread through contaminated meat products and make treatment of infections harder to manage. Therefore, continued monitoring of *Salmonella* serotypes, phenotypic and genotypic resistance, mobile resistance elements, and virulence genes remain important [6,32,33]. 

This study has some limitations. First, the number of isolates was relatively small, and all isolates were recovered from retail chicken meat. Therefore, the findings reflect the characteristics of the analyzed isolate collection and should not be generalized to other poultry products, production systems, geographic regions, or the broader poultry industry in Türkiye. Second, phenotypic colistin susceptibility testing was not performed, and the molecular analysis was restricted to selected antimicrobial resistance and virulence genes. Therefore, additional determinants, including other *mcr* variants (*mcr6–mcr10*), chromosomal mutations, efflux-related mechanisms, and other mobile genetic elements, may not have been detected.

## 4. Conclusions

In conclusion, *S.* Infantis was the most common *Salmonella* serotype detected among our isolates originating from retail chicken meat samples. Furthermore, quinolone resistance in *S.* Infantis was more prevalent than in *S.* Enteritidis. The distribution of the investigated virulence genes indicates that these isolates possess genetic determinants associated with pathogenic potential. In addition, the frequent detection of the *int1* gene warrants further investigation into integron-associated resistance determinants and their contribution to the epidemiology of antimicrobial resistance in *Salmonella*. While *mcr1*–*mcr5* genes were not detected in our isolates and PMQR genes were identified at a low frequency in *S.* Infantis isolates, our findings suggest that retail chicken meat can serve as a significant reservoir of antimicrobial-resistant *Salmonella* and potentially virulent strains. The study results emphasize the importance of foodborne pathogen surveillance as well as enhanced control measures in poultry production and food safety systems.

## 5. Methods

### 5.1. Salmonella Isolates and Serotyping

The *Salmonella* isolates analyzed in this study were obtained from the bacterial culture collection of the Department of Food Hygiene and Technology, Faculty of Veterinary Medicine, Dicle University (Diyarbakir, Türkiye). A total of 53 previously identified *Salmonella* isolates recovered from retail chicken meat in our earlier studies [41,42], using the isolation procedure described by Yoo et al. (2014) [43], were included in the present study. Detailed information on the sampling design, number of retail chicken meat samples, sampling period, geographical coverage, and retail outlets has been reported in the original studies [41,42]. As the present study focused on the phenotypic and molecular characterization of previously identified isolates rather than estimating the prevalence or epidemiological distribution of *Salmonella* in retail chicken meat, these sampling details are not repeated here. Only one *Salmonella* isolate recovered from each positive chicken meat sample was included in the present study. The isolates were stored at −80 °C in tryptic soy broth (Neogen, Lancashire, UK) supplemented with 20% glycerol until use. After thawing, the isolates were revived in tryptic soy broth and incubated at 37 °C for 18–24 h prior to serotyping, antimicrobial susceptibility testing, and molecular analyses. The serotyping analysis of the *Salmonella* isolates was performed at the Department of Microbiology, Faculty of Veterinary Medicine, Ankara University (Ankara, Türkiye), according to the Kauffmann–White scheme [44]. 

### 5.2. Antimicrobial Susceptibility Testing

The Kirby-Bauer disk diffusion method was used to test the susceptibility to quinolone antibiotics of the *Salmonella* isolates on Mueller Hinton agar (Oxoid, Hampshire, UK). The isolates were tested for susceptibility to enrofloxacin (5 µg), norfloxacin (10 µg), ciprofloxacin (5 µg) and moxifloxacin (5 µg). The results were interpreted according to the Clinical and Laboratory Standards Institute [45] guidelines for enrofloxacin, and the European Committee on Antimicrobial Susceptibility Testing (EUCAST) guidelines, version 14.0 [10], for norfloxacin, ciprofloxacin, and moxifloxacin.

Following disk diffusion testing, the minimum inhibitory concentration (MIC) of ciprofloxacin was determined using the broth microdilution method in sterile 96-well microplates as previously described [46]. Briefly, *Salmonella* isolates were cultured overnight in Mueller–Hinton broth (MHB, Neogen, Lancashire, UK), and the bacterial suspension was adjusted to approximately 1 × 10^6^ CFU/mL. Two-fold serial dilutions of ciprofloxacin were prepared in MHB to obtain final concentrations ranging from 1 to 0.02 μg/mL. Subsequently, 10 μL of the standardized bacterial suspension was inoculated into each well containing 100 μL of the corresponding ciprofloxacin dilution, resulting in a final inoculum of approximately 1 × 10^5^ CFU/mL. The microplates were incubated at 37 °C for 18–24 h, and the turbidity was observed.

### 5.3. DNA Extraction and PCR Confirmation

The genomic DNA of 53 *Salmonella* isolates was extracted using a boiling method [47]. The isolates serotyped as *S*. Infantis, and *S*. Enteritidis were confirmed by the conventional PCR method using species-specific and serotype-specific primers [47]. Information about sequences and primers is presented in Table 7. PCR amplifications were performed containing 1 μL forward primer (10 pmol) (Sentromer DNA Technologies, İstanbul, Türkiye), 1 μL reverse primer (10 pmol) (Sentromer DNA Technologies, Türkiye), 0.5 μL dNTPs (10 mM dNTP mix; Thermo Fisher Scientific, Vilnius, Lithuania), 3 μL MgCl_2_ (25 mM, Thermo Fisher Scientific, Vilnius, Lithuania), 2.5 μL PCR reaction buffer (10×, Thermo Fisher Scientific, Vilnius, Lithuania) 0.2 μL Taq DNA polymerase (Thermo Fisher Scientific, Vilnius, Lithuania), 2 µL of DNA and nuclease-free water to a final volume of 25 µL. PCR amplification for *invA* gene, *S*. Infantis (878f-1275r) and *S.* Enteritidis (ENTF-ENTR) was carried out in T100^TM^ Thermal Cycler (Bio-Rad, Singapore) as follows: 94 °C initial denaturation for 3 min, 30 cycles of 94 °C for 1 min, 54 °C for 60 s (64 °C for *S.* Enteritidis), 72 °C for 1 min, and a final extension at 72 °C for 7 min. The amplification for *S*. Infantis (558f-1275r) was carried out as follows: 94 °C initial denaturation for 5 min, 30 cycles of 94 °C for 1 min, 65 °C for 45 s, 72 °C for 1 min, and a final extension at 72 °C for 7 min. The amplified PCR products were analyzed by electrophoresis on 1.5% agarose gel (Prona Agarose Biomax, Burgos, Spain) containing SafeView Classic (Applied Biological Materials, Richmond, BC, Canada) at 100 V for 60 min. The amplified PCR products were visualized using a gel imaging system (Vilber Lourmat, Collégien, France). Positive strains (*S*. Enteritidis ATCC 13076 and *S*. Typhimurium ATCC 700408) were obtained from the strain collection of Ankara University Faculty of Veterinary Medicine Department of Microbiology and Dicle University Faculty of Veterinary Medicine Department of Food Hygiene and Technology.

### 5.4. Detection of Class 1 Integron, Antibiotic Resistance and Virulence Genes

Class 1 integrons were investigated by the detection of the *int1* gene [16]. The PCR amplification protocol was the same as the protocol used for detection of the *invA* gene. The primer and sequence information for the antibiotic resistance genes are shown in Table 7. PCR amplification was performed using 0.5 µM of each primer, 0.2 mM dNTPs, 2 mM MgCl_2_, 1× PCR reaction buffer, 1U of Taq DNA, and nuclease-free water to a final volume of 50 µL. The amplification was performed as follows: strand separation at 95 °C for 3 min, followed by 35 cycles of 95 °C for 30 s, 50 °C for 30 s, and 72 °C for 1 min. Finally, there was a 2 min extension at 72 °C for further strand extension. The presence of genes encoding virulence factors was investigated by the determination of 11 virulence genes (Table 7). The PCR amplification protocol was the same as the protocol used for the detection of the *invA* gene and class 1 integron. However, the PCR amplification was performed at different annealing temperatures for *sipA*, *sopD*, *sopB*, *sifA* and *sitC* (61 °C), *spvC* (51 °C) and *sopE* (65 °C) primers [48]. The amplification for the *pefA* gene was carried out as follows: 95 °C initial denaturation for 5 min, 25 cycles of 94 °C for 30 s, 66,5 °C for 30 s, 72 °C for 2 min, and a final extension at 72 °C for 10 min [49]. Electrophoresis and imaging of all amplified PCR products were performed as mentioned above.

**Table 7:** Target genes, primer sequences (5′–3′), amplicon sizes, and references.

Amplification Target	(5′–3′) Primer Sequences	Primer	Amplicon Size (bp)	References
*S*. Infantis (*fljB* gene)	F-AACAACGACAGCTTATGCCG	558f-1275r	727	[50]
	R-CCACCTGCGCCAACGCT			
	F-TTGCTTCAGCAGATGCTAAG	878f-1275r	413	
	R-CCACCTGCGCCAACGCT			
*S.* Enteritidis	F-TGTGTTTTATCTGATGCAAGAGG	ENT	304	[51]
	R-TGAACTACGTTCGTTCTTCTGG			
*Antibiotic resistance genes*				
Class 1 integron (Integrase1)	F-GCCTTGCTGTTCTTCTAC	Int1-F/B	558	[16]
	R-GATGCCTGCTTGTTCTAC			
*mcr1*	F-CGGTCAGTCCGTTTGTTC	CLR5	1626	[22]
	R-CTTGGTCGGTCTGTAGGG			
*mcr2*	F-TGTTGCTTGTGCCGATTGG	mcr-2	1617	[52]
	R-AGATGGTATTGTTGGTTGCTG			
*mcr3*	F-TTGGCACTGTATTTTGCATTT	mcr-3	542	[53]
	R-TTAACGAAATTGGCTGGAACA			
*mcr4*	F-AATTGTCGTGGGAAAAGCCGC	mcr4	1062	[54]
	R-CTGCTGACTGGGCTATTACCGTCAT			
*mcr5*	F-GTGAAACAGGTGATCGTGACTTACCG	mcr5	271	
	R-CGTGCTTTACACCGATCATGTGCT			
*qnrA*	F-ATTTCTCACGCCAGGATTTG	qnrA	516	[55]
	R-GATCGGCAAAGGTTAGGTCA			
*qnrB*	F-GATCGTGAAAGCCAGAAAGG	qnrB	469	
	R-ATGAGCAACGATGCCTGGTA			
*qnrC*	F-GGGTTGTACATTTATTGAATCG	qnrC	307	[56]
	R-CACCTACCCATTTATTTTCA			
*qnrSm*	F-GCAAGTTCATTGAACAGGGT	qnrSm	428	[57]
	R-TCTAAACCGTCGAGTTCGGCG			
*Virulence genes*				
*invA*	F-GTGAAATTATCGCCACGTTCGGGCAA	invA	284	[58]
	R-TCATCGCACCGTCAAAGGAACC			
*sipA*	F-ATGGTTACAAGTGTAAGGACTCAG	sipA	2055	[59]
	R-ACGCTGCATGTGCAAGCCATC			
*sipD*	F-ATGCTTAATATTCAAAATTATTCCG	sipD	1029	
	R-TCCTTGCAGGAAGCTTTTG			
*sopD*	F-GAGCTCACGACCATTTGCGGCG	sopD	1291	[60]
	R-GAGCTCCGAGACACGCTTCTTCG			
*sopB*	F-GCTCTAGACCTCAAGACTCAAGATG	sopB	1987	
	R-GCGGCCGCTACGCAGGAGTAAATCGGTG			
*sopE*	F-ATTGTTGTGGCGTTGGCATCGT	sopE	376	[61]
	R-AATGCGAGTAAAGATCCGGCCT			
*sopE2*	F-TACTACCATCAGGAGG	sopE2	995	[60]
	R-GAATGTTTTATGTGACGCAG			
*sitC*	F-CAGTATATGCTCAACGCGATGTGGGTCTCC	sitC	768	[49]
	R-CGGGGCGAAAATAAAGGCTGTGATGAAC			
*ssaR*	F-GTTCGGATTCATTGCTTCGG	ssaR	1628	[62]
	R-TCTCCAGTGACTAACCCTAACCAA			
*sifA*	F-ATGCCGATTACTATAGGCAATGG	sifA	1011	
	R-TTATAAAAAACAACATAAACAGCCG			
*spvC*	F-ACTCCTTGCACAACCAAATGCGGA	spvC	571	[63]
	R-TGTCTCTGCATTTCGCCACCATCA			
*pefA*	F-GCGCCGCTCAGCCGAACCAG	pefA	157	[49]
	R-GCAGCAGAAGCCCAGGAAACAGTG			

### 5.5. Statistical Analysis

Statistical analyses were conducted using IBM SPSS Statistics version 24 (IBM Corp., Armonk, NY, USA). Comparisons of antimicrobial resistance profiles, class 1 integron prevalence, and virulence gene distributions between *Salmonella* Infantis and *Salmonella* Enteritidis isolates were performed using Fisher’s exact test. Statistical significance was defined as *p* < 0.05.

## Figures and Tables

**Table 1:** Phenotypic resistance of *S.* Infantis and *S*. Enteritidis isolates to quinolone antimicrobials.

	Number of Resistant Isolates [*n* (%)]			
	Antibiotics			
Serotypes	Enrofloxacin	Norfloxacin	Ciprofloxacin	Moxifloxacin
*S*. Infantis (41)	40 (97.56)	24 (58.54)	32 (78.05)	26 (63.41)
*S*. Enteritidis (12)	7 (58.33)	0	0	0
Total (53)	47 (88.67)	24 (45.28)	32 (60.37)	26 (49.05)

**Table 2:** Quinolone resistance patterns of *S.* Infantis and *S*. Enteritidis isolates.

Pattern	Antibiotic Patterns	Number of Resistant Isolates [*n* (%)]		
		*S*. Infantis (40)	*S*. Enteritidis (7)	Total (47)
A	Enrofloxacin, Norfloxacin, Ciprofloxacin, Moxifloxacin	22 (55)	0	22 (46.8)
B	Enrofloxacin	6 (15)	7 (100)	13 (27.65)
C	Enrofloxacin, Ciprofloxacin	6 (15)	0	6 (12.76)
D	Enrofloxacin, Ciprofloxacin, Moxifloxacin	3 (7.5)	0	3 (6.38)
E	Enrofloxacin, Norfloxacin	1 (2.5)	0	1 (2.12)
F	Enrofloxacin, Moxifloxacin	1 (2.5)	0	1 (2.12)
G	Enrofloxacin, Norfloxacin, Ciprofloxacin	1 (2.5)	0	1 (2.12)

**Table 3:** Ciprofloxacin susceptibility of *S.* Infantis and *S.* Enteritidis determined by the broth microdilution method [*n* (%)].

Serotypes	S	I	R
*S*. Infantis (41)	0	40 (97.56)	1 (2.44)
*S*. Enteritidis (12)	11 (91.67)	1 (8.33)	0
Total (53)	11 (20.75)	41 (77.35)	1 (1.88)

S: susceptible, I: intermediate, R: resistance.

**Table 4:** Presence of class 1 integron (*int1*) gene in *S.* Infantis and *S.* Enteritidis [*n* (%)].

Serotypes	No. of Class 1 Integron Gene Positive Isolates	No. of Class 1 Integron Gene Negative Isolates
*S*. Infantis (41)	33 (80.49)	8 (19.51)
*S*. Enteritidis (12)	11 (91.67)	1 (8.33)
Total (53)	44 (83.01)	9 (16.98)

**Table 5:** Distribution of virulence-associated genes among *S.* Infantis and *S.* Enteritidis isolates.

Serotypes	Virulence Genes										
	*sipA*	*sipD*	*sopD*	*sitC*	*ssaR*	*sifA*	*sopE*	*sopE2*	*sopB*	*spvC*	*pefA*
*S*. Infantis (41)	30 (73.17)	32 (78.05)	33 (80.49)	41 (100)	15 (36.58)	9 (21.95)	40 (97.56)	0	34 (82.93)	0	0
*S*. Enteritidis (12)	11 (91.67)	0	11 (91.67)	12 (100)	1 (8.33)	1 (8.33)	12 (100)	12 (100)	4 (33.33)	7 (58.33)	7 (58.33)
Total (53)	41 (77.35)	32 (60.37)	44 (83.01)	53 (100)	16 (30.18)	10 (18.86)	52 (98.11)	12 (22.64)	38 (71.69)	7 (13.2)	7 (13.2)

**Table 6:** Virulence gene profiles of *S.* Infantis and *S.* Enteritidis isolates.

Profile	Virulence Genes											Serotypes [*n* (%)]		
	*sipA*	*sipD*	*sopD*	*sitC*	*ssaR*	*sifA*	*sopE*	*sopE2*	*sopB*	*spvC*	*pefA*	*S*. Infantis (41)	*S*. Enteritidis (12)	Total (53)
1.	+	+	+	+	−	−	+	−	+	−	−	12 (29.27)	0	12 (22.64)
2.	+	+	+	+	+	−	+	−	+	−	−	7 (17.07)	0	7 (13.20)
3.	+	+	+	+	+	+	+	−	+	−	−	5 (12.2)	0	5 (9.43)
4.	+	−	+	+	−	−	+	+	−	−	−	0	3 (25)	3 (5.66)
5.	+	−	+	+	−	−	+	+	+	+	+	0	3 (25)	3 (5.66)
6.	+	−	+	+	−	−	+	+	−	+	+	0	2 (16.67)	2 (3.77)
7.	−	−	−	+	−	−	+	−	+	−	−	2 (4.88)	0	2 (3.51)
8.	−	−	+	+	−	−	+	−	+	−	−	2 (4.88)	0	2 (3.51)
9.	+	+	−	+	−	−	+	−	−	−	−	1 (2.44)	0	1 (1.88)
10.	+	−	+	+	−	+	+	+	−	+	+	0	1 (8.33)	1 (1.88)
11.	+	−	+	+	−	+	+	+	+	+	+	0	1 (8.33)	1 (1.88)
12.	+	−	+	+	+	−	+	+	−	−	−	0	1 (8.33)	1 (1.88)
13.	+	+	+	+	−	+	+	−	+	−	−	1 (2.44)	0	1 (1.88)
14.	−	−	−	+	−	−	+	+	−	−	−	0	1 (8.33)	1 (1.88)
15.	+	−	+	+	−	−	+	−	+	−	−	1 (2.44)	0	1 (1.88)
16.	+	+	−	+	−	−	+	−	+	−	−	1 (2.44)	0	1 (1.88)
17.	−	−	−	+	+	−	+	−	−	−	−	1 (2.44)	0	1 (1.88)
18.	−	−	−	+	+	−	+	−	+	−	−	1 (2.44)	0	1 (1.88)
19.	−	−	+	+	−	−	+	−	−	−	−	1 (2.44)	0	1 (1.88)
20.	−	+	−	+	−	−	+	−	−	−	−	1 (2.44)	0	1 (1.88)
21.	+	−	+	+	−	+	+	−	+	−	−	1 (2.44)	0	1 (1.88)
22.	−	+	+	+	−	−	+	−	−	−	−	1 (2.44)	0	1 (1.88)
23.	−	+	+	+	−	−	+	−	+	−	−	1 (2.44)	0	1 (1.88)
24.	+	+	+	+	+	+	−	−	+	−	−	1 (2.44)	0	1 (1.88)
25.	−	+	−	+	−	+	+	−	−	−	−	1 (2.44)	0	1 (1.88)

+, presence of the corresponding virulence gene; −, absence of the corresponding virulence gene.

## Data Availability

The original contributions presented in this study are included in the article. Further inquiries can be directed to the corresponding author.
